# Underage alcohol policies across 50 California cities: an assessment of best practices

**DOI:** 10.1186/1747-597X-7-26

**Published:** 2012-06-26

**Authors:** Sue Thomas, Mallie J Paschall, Joel W Grube, Carol Cannon, Ryan Treffers

**Affiliations:** 1 Pacific Institute for Research and Evaluation, P.O. Box 7042, Santa Cruz, CA, 95061, USA; 2 Prevention Research Center, 1995 University Ave, Suite 450, Berkeley, CA, 94704, USA

**Keywords:** Alcohol policy, Alcohol laws, Alcohol deterrents, Youth drinking, Public policy, Health policy

## Abstract

**Background:**

We pursue two primary goals in this article: (1) to test a methodology and develop a dataset on U.S. local-level alcohol policy ordinances, and (2) to evaluate the presence, comprehensiveness, and stringency of eight local alcohol policies in 50 diverse California cities in relationship to recommended best practices in both public health literature and governmental recommendations to reduce underage drinking.

**Methods:**

Following best practice recommendations from a wide array of authoritative sources, we selected eight local alcohol policy topics (e.g., conditional use permits, responsible beverage service training, social host ordinances, window/billboard advertising ordinances), and determined the presence or absence as well as the stringency (restrictiveness) and comprehensiveness (number of provisions) of each ordinance in each of the 50 cities in 2009. Following the alcohol policy literature, we created scores for each city on each type of ordinance and its associated components. We used these data to evaluate the extent to which recommendations for best practices to reduce underage alcohol use are being followed.

**Results:**

(1) Compiling datasets of local-level alcohol policy laws and their comprehensiveness and stringency is achievable, even absent comprehensive, on-line, or other legal research tools. (2) We find that, with some exceptions, most of the 50 cities do not have high scores for presence, comprehensiveness, or stringency across the eight key policies. Critical policies such as responsible beverage service and deemed approved ordinances are uncommon, and, when present, they are generally neither comprehensive nor stringent. Even within policies that have higher adoption rates, central elements are missing across many or most cities’ ordinances.

**Conclusion:**

This study demonstrates the viability of original legal data collection in the U.S. pertaining to local ordinances and of creating quantitative scores for each policy type to reflect comprehensiveness and stringency. Analysis of the resulting dataset reveals that, although the 50 cities have taken important steps to improve public health with regard to underage alcohol use and abuse, there is a great deal more that needs to be done to bring these cities into compliance with best practice recommendations.

## Background

The U.S. Healthy People 2010 objectives, a set of science-based, ten year national objectives to improve the health of Americans, included reducing underage alcohol use, especially among younger adolescents [1]. These objectives were based on the 1979 U.S. Surgeon General’s Report, *Healthy People: The Surgeon General’s Report on Health Promotion and Disease Prevention*, as well as objectives from previous Healthy People initiatives in 1990 and 2000 and were prepared by experts from multiple federal agencies, offered for public comments, and finalized by a Federal Interagency Workgroup.

To help accomplish the goal of reducing underage alcohol use, a wide array of authoritative best practice recommendations pertaining to federal, state, and local laws has emerged, including recommendations from the *U.S. Surgeon General’s 2007 Call to Action to Prevent and Reduce Underage Drinking*[2] and *The Community Guide*, developed by The Community Preventive Services Task Force, an independent body of public health and prevention experts supported by The U.S. Centers for Disease Control and Prevention [3].

Epidemiological studies indicate that the prevalence of past-year and past-30-day alcohol use among underage youth in the U.S. has declined very little since the late 1990s [4]. According to the 2010 Monitoring the Future survey (a long-term epidemiological survey of trends in licit and illicit drug use among Americans funded by the U.S. National Institute on Drug Abuse), alcoholic beverages are among the most widely used psychoactive substances by American young people. For example, in 2010, the proportions of 8th, 10th, and 12th graders who reported drinking at least one alcoholic beverage in just the 30-day period prior to the survey were 14%, 29%, and 41%, respectively. Binge drinking rates (defined as consuming five or more drinks in a row) for a prior two week interval were 7.2%, 16.3%, and 23.2% for grades 8, 10, and 12, respectively. And perceived availability was at very high levels: 90% of twelfth graders reported that it would be fairly easy or very easy for them to get alcohol [5].

The harms associated with underage alcohol use are extensive and include youth-drunk driving crashes and fatalities. Additional harms related to underage drinking include other unintentional injuries such as poisonings, drownings, falls, and burns; alcohol-related suicides, homicides, rapes, robberies, and other assaults; risky sexual activity; and longer-term physical and emotional impairments resulting from alcohol use and abuse [6-9]. Finally, the annual social cost of underage drinking in the U.S. in 2006 has been reported by the Centers for Disease Control (CDC) to be $224 billion or approximately $1.90 per drink [10]. In light of these statistics, preventing drinking and drinking problems among youth remains a high priority.

### Alcohol policy: the importance of the law

In the U.S., policies pertaining to the manufacture, sale, and use of alcohol are established by the federal, state, and local governments. The legal basis for federal and state regulation of alcohol comes from the U.S. Constitution. The 21st Amendment (1933) repealed prohibition and granted states power to regulate alcoholic beverages by permitting them to determine the rules of importation or sale; the structure of distribution; and alcohol sale and possession. Nevertheless, the 21^st^ Amendment does not preclude the federal government from regulating alcohol via the Commerce Clause of the Constitution which grants Congress the authority to regulate commerce among the states. Additionally, Congress can use its constitutional taxing power to assess and collect alcoholic beverage taxes. Finally, the federal government can influence state alcohol policy by offering financial incentives for states to enact certain types of laws. This is why all U.S. states currently prohibit underage purchase and consumption of alcohol. Had they not enacted such laws, states would have forfeited a portion of their federal highway funding. With respect to local government control of alcohol policy, states vary in the nature of the authority allocated to them. In many states, local governments enact laws (ordinances) to regulate the sale and distribution of alcohol within their jurisdictions. In other states, alcohol control is retained at the state level with little or no leeway accorded to localities [9].

Federal, state, and local policy approaches to prevention are frequently advocated as among the best tools available to reduce youth access to alcohol, drinking, and drinking problems in the U.S. [11-13]. Broadly defined, alcohol policy includes the following components: formal legal and regulatory mechanisms, rules, and procedures for reducing the consumption of alcohol or risky drinking behaviors, and enforcement of these measures [14-17]. The purpose of such policies is to increase the costs to young people for obtaining, possessing, and consuming alcohol, and to adults for providing alcohol to minors. Such policies may also reinforce community norms against underage drinking and against providing alcohol to youth.

Typically, studies of alcohol policy effectiveness among youth in the United States focus on the federal and state levels, especially on the effects of the 1984 National Minimum Drinking Age Act (MLDA), which required states to enact a minimum age of 21 years for purchase or public possession of alcohol or risk losing federal highway funds. Since 1987, the minimum legal drinking age has been 21 years of age in all 50 states and the District of Columbia. A broad range of studies illuminates the fact that increasing the MLDA significantly decreases drinking and drinking problems among young people [18-22].

Yet, as is the case with any set of policies, including the MLDA, those intended to reduce underage drinking and the harms associated with it vary widely as to their presence, specificity, and comprehensiveness. Arguably, ceteris paribus, having a policy is better than having no policy, but weak ones are likely to have fewer positive effects than strong policies.

In contrast to a nuanced theoretical and practice approach to studying underage or adult drinking laws, the majority of prior studies on the effects of alcohol control policies have used “presence/absence” indicators of policies rather than measures reflecting policy comprehensiveness (number of provisions) and stringency (restrictiveness). Still, a few recent studies have attempted to address this limitation. Importantly, [Brand et al. 23] developed an Alcohol Policy Index (API) to gauge the strength of alcohol control policies in 30 countries based on 16 policy topics comprising five domains: alcohol availability, drinking context, price, advertising, and motor vehicles. The authors found that the overall API was inversely associated with per capita alcohol consumption in the 30 countries. A more recent study by[ Paschall, Grube and Kypri 24] found that the overall API score, and the alcohol availability domain rating in particular, were inversely related to national prevalence rates of any past-30-day alcohol use and frequent alcohol consumption among adolescents.

At the U.S. state level, [Fell et al. 25,^26^] examined relationships between comprehensiveness and stringency ratings of an array of state alcohol policies and traffic crashes and fatalities among underage drivers (< 21 years old). Results indicated that stronger laws prohibiting underage possession and purchase were associated with an 11.2% reduction over time in the ratio of underage drinking to nondrinking drivers in fatal traffic crashes, and stronger laws restricting the use of fake identifications by minors were associated with a 7.3% decrease in the percentage of underage drivers under the influence of alcohol in fatal crashes. More recently, [Ringwalt and Paschall 27] examined associations between U.S. state beer keg registration policy ratings and state-level prevalence estimates of past-30-day binge drinking, driving after drinking, and riding with a drinking driver among adolescents. Correlational analyses indicated significant inverse associations between more comprehensive and stringent keg registration policies and all three of these behaviors.

Internationally, a systematic review of 33 evaluations of minimum drinking age (MLDA) laws in the United States, Canada, and Australia found a median decline of 16% in crash-related outcomes for the targeted age groups following increases in the MLDA [28]. Conversely, increases in alcohol-related traffic crash injuries and traffic crash hospitalizations among youth were found after the drinking age in New Zealand was lowered from 20 to 18 years [29].

Much less research exists on the effectiveness of local level policy efforts–despite the fact that local communities may be particularly important for policy interventions. Although alcohol control is primarily a state responsibility in the U.S., many states permit cities and counties to enact alcohol ordinances that are more restrictive than those required by state law as long as they don’t contradict those laws. In California, the site of the research reported here, the state Constitution provides that the state has “the exclusive right and power to license and regulate the manufacture, sale, purchase, possession and transportation of alcoholic beverages.” [30].

Within the constitutional context, the State Alcoholic Beverage Control Act specifies the types of alcohol outlets and licenses, restricting their location and number to some degree, and providing minimum standards for operation. Nonetheless, California localities can regulate alcohol as both the state legislature and the courts recognize that interests of cities and counties overlap with state interests. Using local zoning, land use, and police powers, cities and counties may enact a range of ordinances and regulations such as, but not limited to, conditional use permit policies, deemed approved ordinance policies, and responsible beverage service (RBS) training policies. The extent to which elements of these policies may apply to new or existing alcohol outlets is also prescribed by California state law: greater latitude is accorded to localities with respect to new outlets than to outlets that existed prior to enactment of any local ordinance or regulation (see, for example, Ventura County Behavioral Health Department Publication 2007 [31]). Despite restrictions on what localities in California may enact with regard to the regulation of alcohol, localities have a host of policy tools at their disposal. Hence, local policy enactment is a potentially important tool for reducing underage drinking and drinking problems.

Extant local-level research indicates that there is considerable variability in alcohol policy and enforcement directed at adults and minors [32-36]. In a study focusing specifically on California communities, Wittman and Hilton analyzed the experiences of these communities’ preventive use of local alcohol-related ordinances in the 1980s [37]. Their 1984 survey of planning directors in all California cities with planning and zoning departments revealed relatively high levels of concern regarding alcohol outlet problems, but lower regulation activity generally. For example, conditional use permits (CUPs) for on-sale only outlets that regulate outlet distance from schools, hours of sale, and adequate lighting (among other policy elements), were present in only 29% of cities and 30% for on- and off-sale outlets. This type of outlet regulation was most common in larger cities and in communities with higher levels of urbanicity. It is clear from this survey that much more was needed to achieve greater reductions in the harms associated with alcohol use and abuse, including those found among minors.

To the extent that research has addressed issues of effectiveness of local alcohol policy ordinances on reductions in drinking and its associated harms, available findings, some cross-sectional and some longitudinal, generally indicate a positive relationship between policy and public health improvements. For example, several studies have found that restricting outlet density can affect alcohol consumption and problems [38-44]. In one of the few studies focused on outlet density and its effects on youth drinking, [45] outlet density was found to be positively related to frequency of driving after drinking and riding with drinking drivers among 16 to 20-year-old youth.

Recent systematic reviews suggest that other local policies, when sufficiently implemented and enforced, can be effective in reducing excessive alcohol consumption and related harms. Potentially effective local policies include dram shop liability [46,47], restrictions on hours and days of alcohol sales, [48,49] and restrictions on outlet density [50].

A number of studies have investigated the effectiveness of responsible beverage service training programs (RBS) [51-53]. [Paschall et al. 52] found that outlets participating in Oregon’s Responsible Vendor Program, a comprehensive ]program that includes RBS, were less likely to sell alcohol to underage-appearing buyers than non-participating outlets. Other studies indicate that RBS training can increase checks on age identification and reduce alcohol sales to minors [54]. On the other hand, a systematic review of studies on RBS concluded that, although there was some evidence that interventions in bars could reduce bar staff injuries and possibly aggression, there was not sufficient evidence to ascertain that these interventions reduce drinking-related injuries overall [55].

Despite findings suggesting that local alcohol policies can be effective generally in reducing alcohol availability to youth, underage drinking and drinking problems in California and in other locations, there is a dearth of research addressing the extent to which local jurisdictions have enacted a broad range of recommended policies, and the degree to which those policies conform to best practices standards in both public health literature and governmental calls to action. As a result, extant research may underestimate the effectiveness of policy approaches. These are important considerations as weak laws may impede the goal of reducing the harms associated with underage drinking.

Accordingly, in the study presented here, we sought to expand the scope of research on local-level alcohol laws directed toward reduction in youth drinking in two ways. First, we tested a methodology and created an original, comparative dataset on local-level alcohol policy ordinances in California across multiple policy topics. Most of the research on the local level, either in California or elsewhere, has not focused on widespread data collection that includes multiple cities and multiple types of policy. This is likely due to the fact that, unlike for state or federal laws in the U.S., no comprehensive, reliable, searchable database currently exists for local laws across the nation. The extent to which reliable and valid alternatives can be found improves the ability for researchers to test theories of the importance of policy adoption to reduction of underage drinking.

Our second goal was to evaluate the presence, comprehensiveness (number of provisions), and stringency (restrictiveness) of eight local alcohol policies in 50 diverse California cities. These eight policies (conditional use permits for new establishments selling or serving alcohol [CUPs], deemed approved ordinances [DAOs] for existing establishments selling or serving alcohol, regulations on outdoor advertising/billboards of alcoholic beverages, regulations on public drinking, responsible beverage service [RBS] training requirements, social host policies, special outdoor events policies, and regulations on window advertising), were chosen to represent the range of available local efforts as well as to reflect a wide array of authoritative best practice recommendations to reduce underage drinking. The choice of California rested on the size and diversity of the state as well as the comparatively large amount of prior local-level research focused there, upon which this study could build.

## Methods: data sources and policy ratings

Our sources of authoritative best practice recommendations to reduce underage drinking included the U.S. Surgeon General’s 2007 Call to Action to Prevent and Reduce Underage Drinking; the Community Guide developed by The Community Preventive Services Task Force [3], an independent body of public health and prevention experts (supported by The U.S. Centers for Disease Control and Prevention); a host of model ordinances; and peer-review policy research pertaining to the effects of existing state and federal law (see Table 1 for complete list of sources). The review of these sources resulted in the selection of eight local alcohol policy topics and helped us determine the presence or absence as well as the stringency (restrictiveness) and comprehensiveness (number of provisions) of each ordinance in each city in 2009. These policies are as follows and are explained in some detail in Table 2; the specific policy elements within each policy topic appear in Table 3:

· Conditional use permits (CUPs) required for new establishments selling or serving alcohol that regulate such conditions as hours of operation, types of alcoholic beverages that can be served, and outdoor lighting requirements;

· Deemed approved ordinances (DAOs) for preexisting establishments selling or serving alcohol;

· Outdoor advertising/billboards of alcoholic beverages ordinances;

· Public drinking ordinances;

· Responsible beverage service (RBS) training required for staff of establishments selling or serving alcohol;

· Social host policies mandating criminal and/or civil sanctions of hosts of underage drinking parties;

· Special outdoor events policies governing alcohol service and consumption at such events such as street fairs;

· Window advertising of alcoholic beverages provisions.

**Table 1:** Local policies and their definitions

	
CONDITIONAL USE PERMITS (CUPs)	In California, state government has the exclusive right over alcohol sales licenses. However, local governments have authority to regulate land use to protect health, welfare and safety. To reduce problems related to the density of alcohol outlets (noise, loitering, vandalism, littering, assault and battery, underage purchasing of alcohol, and more), localities use CUPs to regulate retail alcohol sales. CUPs may require that licensed establishments be a minimum distance from schools, parks, or churches; limit alcohol sales to certain hours; maintain nighttime lighting; and take action to prevent nuisance, and criminal activities on or in close proximity to the premises.
DEEMED APPROVED ORDINANCES (DAOs)	DAOs use the same zoning authority to apply CUP-equivalent standards to pre-existing alcohol outlets. Outlets in existence at the time CUPs have been enacted are exempted from CUP requirements. However, DAOs require these pre-existing outlets to meet the same types of standards as those governed by CUPs.
RESPONSIBILE BEVERAGE SERVICE TRAINING (RBS)	RBS Training ordinances establish mandatory or voluntary compliance with merchant education and server action standards to ensure compliance with prohibitions on serving minors or intoxicated persons. These ordinances may require that licensees, managers, servers, or other employees attend RBS training.
PROHIBITIONS ON HOSTING UNDERAGE DRINKING PARTIES (SOCIAL HOST)	Social host ordinances hold individuals responsible for underage drinking events on property they own, lease, or otherwise control. Social host ordinances may be general, applying to all individuals, or they may have provisions that are specific to underage drinking. They are also often closely linked to other laws prohibiting furnishing alcohol to minors, but social host ordinances apply without regard to who furnishes the alcohol. Criminal or civil penalties may apply to social host violations.
LIMITATIONS ON WINDOW ADVERTISING OF ALCOHOL	These ordinances restrict the size and placement of window advertisements in stores by mandating a maximum percentage of total window space that can be covered generally by advertisements or specifically by alcohol ads.
LIMITATIONS ON BILLBOARDS AND OTHER OUTDOOR ALCOHOL ADVERTISING	Policies that ban all outdoor advertising or limit outdoor advertising of alcoholic beverages, particularly ads exposing minors to alcohol messages. Included in these types of ordinances are bans on ads on public transportation, such as trains and buses, bus shelters, parks, billboards, supermarket carts, parking structures, near schools and residential areas, and at community events such as sporting events, concerts, and street fairs.
PROHIBITIONS ON PUBLIC DRINKING	These are crimes defined as consuming or possessing alcoholic beverages in public. Public intoxication ordinances generally do not depend on specific blood alcohol content levels; instead, they rely on the physical possession of alcohol.
RESTRICTIONS ON ALCOHOL AVAILABILITY FOR SPECIAL EVENTS	Alcohol restrictions at special events, such as concerts, street fairs, and sporting events, control the availability and use of alcohol at these venues. Restrictions can include complete bans on consumption, warning signs about the dangers of alcohol consumption, mandates to establish separate drinking areas into which minors are prohibited, mandatory RBS training for servers, alcohol purchase limitations, and retention of security staff.

**Table 2:** Sources for policy and variable selection

**Source**	**Year(s)**
1. The U.S. Surgeon General’s Call to Action to Prevent and Reduce Underage Drinking (Goals 1,2,4 and 6)	2007
2. The Community Guide by The Community Preventive Services Task Force	2011
3. The U.S. Substance Abuse and Mental Health Services Administration (SAMHSA)’s Inter-Agency Coordinating Committee on Preventing Underage Drinking (ICCPUD) Report to Congress on Plans for Combating Underage Drinking [56]	2004
4. The Institutes of Medicine (IOM) Report on Reducing Underage Drinking	2003
5. Model Ordinances on CUPs and DAOs - Pacific Institute for Research and Evaluation (PIRE)[57]	2007; 2008
6. Model Social Host Ordinance (PIRE) [58]	2005
7. Best Practices Reports on Regulatory Strategies for Preventing Youth Access to Alcohol (PIRE/U.S. Office of Juvenile Justice and Delinquency Prevention (OJJDP)	1999
8. Best Practices Report on Using Local Land Use Powers to Prevent Underage Drinking (PIRE/OJJDP)	2000
9. Center on Alcohol Marketing and Youth (CAMY) Report on Alcohol Advertising Laws [59]	2003
10. Myths and Realities about Drinking in America (PIRE/OJJDP [60])	2002
11. Empirical Research Literature on Alcohol Policy at U.S. federal, state, and local levels including: [Barry et al. 2004 61]; [Dent et al. 2005 62];[ Dills 2010 63]; [Fell et al. 2008 , 200925,^26^]; [Forster et al. 1994,1995 32,^64^]; [Freisthler et al. 2003 51]; Grube 2005 [14]; [Grube and Nygaard 2005 16]; [Gruenewald et al. 2002 40]; [Holder et al. 1997 65]; [Laixuthai and Chaloupka 1993 66]; Mosher et al. 2002 [34]; O’ [Malley and Wagenaar 1991 20]; [Paschall et al. 2007 50]; [Stout et al. 2000 67]; [Toomey and Wagenaar 2002 54]; [Treno et al. 2003 45]; [Voas et al. 2003 68]; [Wagenaar et al. 2005 69]; [Whetten-Goldstein et al. 2000 70]; [Wittman and Hilton 1987 and 2007 37]; [Yu 1997 71];Institute for Public Strategies 2003 [Ker and Chinnock 2008 55]; [Shults et al. 2002 28]; [Hahn et al 2010 48]; [Middleton et al. 2010 49]; [Campbell et al. 2009 50].	1987–2010

**Table 3:** Coding categories and scores for eight local policy topics

**Conditional use permits**	
Presence or absence of alcohol-related CUP	1.00
Mandatory limitations on hours of sales	2.00
Minimum distances from public schools and parks or churches	1.00
Conditions regarding night lighting	1.00
Prohibitions in areas of over-concentration, high crime rate, etc.	2.00
DEEMED APPROVED ORDINANCES	
Presence or absence of alcohol-related DA	1.00
Mandatory limitations on hours of sales	2.00
Conditions regarding night lighting	1.00
Prohibitions in areas of over-concentration, high crime rate, etc.	2.00
RESPONSIBLE BEVERAGE SERVICE	
Existence of law	1.00
Mandatory	2.00
Voluntary	1.00
Some must obtain training (licensee, manager, servers)	1.00
All must obtain training	2.00
Applies to on-premises	1.00
Applies to off –premises	1.00
Applies to new establishments	1.00
Applies to existing establishments	1.00
Presence of certification renewal period	1.00
SOCIAL HOST	
Existence of law	1.00
Applies to underage (person or party)	2.00
Civil	2.00
Criminal	1.00
Range of property types (residence, outdoor property)	1.00
Knowledge requirement	−1.00
WINDOW ADVERTISING	
Existence of law	1.00
Any provision about distance from schools or parks	1.00
Applies to some establishments	1.00
Applies to all establishments	2.00
OUTDOOR ADS/BILLBOARDS	
Existence of law	1.00
Prohibited near schools?	1.00
PUBLIC DRINKING	
Existence of law	1.00
Range of types of public spaces (parks, beaches, schools)	1.00
Narrow range of circumstances in which alcohol is permitted	1.00
SPECIAL OUTDOOR EVENTS	
Existence of law	1.00
Controlled alcohol consumption spaces	1.00
Security measures	1.00

Next, to reflect the elements critical to comprehensiveness and stringency of each type of ordinance, we assigned scores to each policy element. These scores are based on a recent coding scheme to assess the strength of 16 U.S. state-level underage drinking laws [25,26]. This scoring system assigns points for policies that deter young people from using alcohol. A value of zero corresponds with a state not having a law on a particular policy topic, and higher values represent stronger laws. Because localities can, in many cases, pass ordinances on the same topics as state governments, this tested system was judged the most appropriate for our purposes. In our adaptation of the scoring process, a city received a +1for each policy topic if it had the relevant type of ordinance in question; a 0 if no such law existed. Then, each element of law was assigned points for comprehensiveness and stringency. Once the coding procedures were finalized, two research scientists independently scored each policy for each city. Table 3 presents the scoring system. It is important to note that because each law differs in the number of provisions assessed (and possible point additions or deductions), the possible high and low scores that are possible vary across the eight types of laws. Thus, the maximum possible number of points for a law does not imply relative importance of that law compared to the other laws. Each law’s point scale is independent, and scores of different policies are not directly comparable.

The cities for which data (municipal ordinances) were collected for each policy topic in each city were chosen as follows. A geographically diverse sample of 50 non-contiguous California cities with populations between 50,000 and 500,000 were chosen. The population parameters reflect the desire to represent the greatest share of the state’s 481 cities and to include variation in population and environmental characteristics typical among cities of this size in the state (see list of selected cities in Table 4 and 5).

**Table 4:** Policy scores per topic per city

**City**	**Conditional use permits**	**Deemed approved ordinances**	**Public drinking**	**Social host**
Antioch	4.00	0.00	3.00	0.00
Bakersfield	1.00	0.00	3.00	6.00
Baldwin Park	3.00	3.00	3.00	3.00
Chico	1.00	0.00	2.00	0.00
Corona	1.00	0.00	3.00	7.00
Davis	6.00	0.00	3.00	7.00
Diamond Bar	1.00	0.00	3.00	0.00
Fairfield	5.00	0.00	3.00	0.00
Folsom	4.00	0.00	3.00	0.00
Fresno	3.00	0.00	3.00	6.00
Gardena	1.00	0.00	2.00	0.00
Hemet	1.00	0.00	1.00	0.00
Huntington Beach	2.00	0.00	3.00	0.00
Huntington Park	5.00	4.00	3.00	0.00
La Mesa	0.00	0.00	3.00	7.00
Lake Forest	0.00	0.00	3.00	0.00
Lancaster	7.00	3.00	3.00	0.00
Livermore	1.00	0.00	1.00	0.00
Merced	4.00	0.00	3.00	5.00
Milpitas	1.00	0.00	3.00	0.00
Modesto	0.00	0.00	3.00	6.00
Napa	7.00	6.00	3.00	7.00
National City	6.00	0.00	3.00	7.00
Orange	6.00	5.00	3.00	5.00
Petaluma	5.00	4.00	3.00	6.00
Pico Rivera	5.00	0.00	3.00	0.00
Rancho Cucamonga	1.00	0.00	3.00	0.00
Redding	0.00	0.00	3.00	0.00
Redlands	1.00	0.00	3.00	0.00
Richmond	7.00	6.00	3.00	0.00
Sacramento	6.00	5.00	3.00	0.00
Salinas	7.00	0.00	3.00	4.00
San Leandro	7.00	3.00	3.00	6.00
San Rafael	1.00	0.00	3.00	6.00
Santa Barbara	0.00	0.00	3.00	6.00
Santa Clarita	2.00	0.00	3.00	0.00
Santa Cruz	5.00	3.00	3.00	6.00
Santa Maria	3.00	0.00	3.00	5.00
Santa Monica	6.00	0.00	3.00	0.00
Santa Rosa	7.00	3.00	3.00	6.00
Simi Valley	4.00	0.00	3.00	6.00
Stockton	4.00	0.00	3.00	0.00
Sunnyvale	7.00	0.00	3.00	7.00
Temecula	5.00	0.00	3.00	0.00
Tracy	0.00	0.00	3.00	0.00
Turlock	3.00	0.00	3.00	5.00
Ventura	5.00	3.00	3.00	7.00
Visalia	4.00	0.00	3.00	0.00
Vista	4.00	3.00	3.00	6.00
Walnut Creek	6.00	0.00	3.00	0.00

**Table 5:** Policy scores per topic per city for outdoor advertising, special outdoor events, window advertising and responsible beverage service ordinances

**City**	**Outdoor advertising**	**Special outdoor events**	**Window advertising**	**Responsible beverage service**
Antioch	0.00	1.00	0.00	0.00
Bakersfield	0.00	1.00	0.00	0.00
Baldwin Park	1.00	0.00	0.00	0.00
Chico	1.00	0.00	3.00	0.00
Corona	1.00	0.00	3.00	0.00
Davis	0.00	1.00	0.00	0.00
Diamond Bar	1.00	1.00	0.00	7.00
Fairfield	2.00	3.00	0.00	0.00
Folsom	0.00	3.00	0.00	0.00
Fresno	1.00	2.00	0.00	0.00
Gardena	2.00	0.00	3.00	0.00
Hemet	2.00	0.00	0.00	0.00
Huntington Beach	0.00	1.00	0.00	0.00
Huntington Park	1.00	1.00	3.00	0.00
La Mesa	1.00	2.00	0.00	0.00
Lake Forest	0.00	1.00	0.00	0.00
Lancaster	2.00	3.00	2.00	7.00
Livermore	2.00	1.00	0.00	0.00
Merced	1.00	1.00	2.00	0.00
Milpitas	0.00	1.00	0.00	0.00
Modesto	0.00	1.00	0.00	0.00
Napa	0.00	2.00	0.00	0.00
National City	1.00	0.00	0.00	7.00
Orange	1.00	1.00	2.00	7.00
Petaluma	0.00	2.00	0.00	10.00
Pico Rivera	1.00	1.00	2.00	0.00
Rancho Cucamonga	0.00	3.00	0.00	0.00
Redding	2.00	1.00	0.00	0.00
Redlands	1.00	2.00	3.00	0.00
Richmond	2.00	1.00	3.00	0.00
Sacramento	0.00	1.00	0.00	0.00
Salinas	1.00	1.00	2.00	8.00
San Leandro	1.00	1.00	2.00	0.00
San Rafael	2.00	1.00	0.00	0.00
Santa Barbara	2.00	1.00	3.00	0.00
Santa Clarita	1.00	1.00	0.00	0.00
Santa Cruz	0.00	1.00	3.00	4.00
Santa Maria	0.00	2.00	0.00	0.00
Santa Monica	0.00	1.00	2.00	6.00
Santa Rosa	0.00	2.00	1.00	8.00
Simi Valley	0.00	1.00	0.00	0.00
Stockton	0.00	2.00	0.00	0.00
Sunnyvale	1.00	1.00	0.00	0.00
Temecula	1.00	3.00	2.00	4.00
Tracy	0.00	2.00	0.00	0.00
Turlock	1.00	2.00	0.00	0.00
Ventura	0.00	1.00	2.00	6.00
Visalia	0.00	2.00	0.00	0.00
Vista	0.00	3.00	0.00	0.00
Walnut Creek	0.00	1.00	0.00	0.00

Second, to gather the legal data, we used a careful two-stage process for locating all extant ordinances across the cities in the first half of 2009. Because original legal research is necessary for accurate, complete, and up-to-date information, and because, unlike the situation for data on state or federal statutes, there are no comprehensive sites for the collection of municipal ordinances on alcohol policy either in California or throughout the United States, policy data were gathered by first locating the website for each city in our sample and determining that city ordinances were available online. Fortunately, that was the case in each of the 50 cities. The second stage of the process was to contact the City Clerk in each city to ensure that the most recent version of all relevant ordinances were posted online and to obtain copies of the newest version of ordinances in instances in which that was not the case. City Clerks also informed us about city-specific practices related to the type of information posted on websites and how often information is updated.

Relying on secondary sources for any of this information, were they available, would not be adequate to the task. Although many advocacy and think tanks offer compendia of the law on various topics, they are rarely sufficient sources of legal data. These compendia are seriously flawed by the absence of a rigorous research and verification process and/or no documentation of the research process and coding conventions used, and often contain significant errors. Similar problems arise with sole reliance on written ordinances or key informants [72,73].

Once all data were available, they were coded, quality control was performed via two coders who independently checked each other’s work as noted above, and the results were stored in an Access database.

## Results: Are California communities implementing best practices? Variation across policy topics and cities

**Figure 1:**
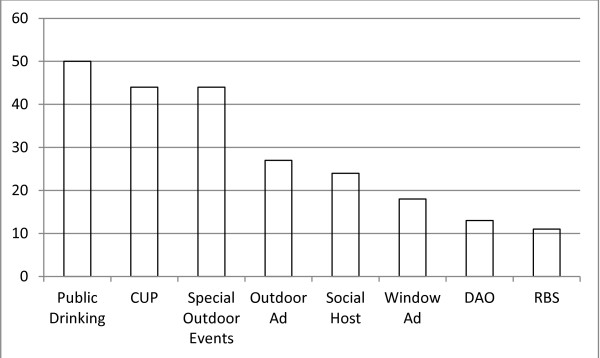
Number of cities in 50 city sample with each type of alcohol policy.

Figure 1 displays the number of cities within the 50 city sample that have each type of policy. This top level analysis reveals considerable variation across policy topics across the cities. For example, although all 50 cities have public drinking prohibitions and the vast majority (44 cities) have both special outdoor events restrictions and CUPs, only 26 cities have outdoor/billboard advertising limitations, and 24 have social host liability ordinances. Fewer than half the cities have window advertising limitations (19), deemed approved ordinances (13), or RBS training requirements (11). The lack of the advertising limitations and deemed approved ordinances may be particularly consequential, as best practices standards in both public health literature and governmental calls to action suggest that these are central to reducing underage drinking and the associated harms [2,3,13,31,74].

### Number of cities in 50 city sample with each type of alcohol policy

Examining provisions within policy topics provides additional insight into the stringency (restrictiveness) and comprehensiveness (number of provisions) of city efforts on each of these policy topics. Table 6 displays frequencies of ordinance provisions per policy topic across the cities. Again, the data reveal substantial variation in adoption of specific provisions within each policy topic across the 50 cities. Best practices recommendations and research literature results indicate that the policy topics most central to reducing underage drinking and its associated harms include conditional use permits/deemed approved ordinances, responsible beverage service training, and social host ordinances. Yet, of the 44 cities with CUP ordinances, only 31 have prohibitions pertaining to new outlets in areas of over-concentration and high crime. The policy literature indicates that this type of provision is extremely important in terms of ordinance efficacy [12,37,74]. Although only 13 of the 50 cities have deemed approved ordinances, all 13 address reduction in crime and violence with special conditions for outlets in areas of high crime. Despite the fact that DAOs apply to existing outlets, these cities include provisions that mandate that outlets operate in a manner consistent with the CUP requirements for new outlets. On the other hand, RBS training ordinances are present in only 11 (20%) of the cities. Specific RBS elements considered to be most important according to best practices [34] include mandates that apply to both new and existing establishments and both on- and off-sale premises. Only 6 cities have requirements that apply to existing establishments, and 10 have requirements that apply to new establishments; only 7 have provisions that apply to off-sale premises, and only 9 have provisions that apply to on-sale premises. Finally, just under half (24) of the 50 cities have enacted social host liability ordinances, and only 21specifically apply to underage parties.

**Table 6:** Frequencies of ordinance provisions per policy topic across the fifty California cities. By number of cities

**Conditional Use Permits**	
Presence of alcohol-related CUP	44
Mandatory limitations on hours of sales	15
Minimum distances from schools, parks, and churches	27
Conditions regarding night lighting	12
Prohibitions in areas of over-concentration, high crime, etc.	31
Deemed Approved Ordinances	
Presence or absence of alcohol-related DAO	13
Mandatory limitations on hours of sales	4
Conditions regarding night lighting	4
Prohibitions in areas of over-concentration, high crime, etc.	13
Responsible Beverage Service Training	
Existence of law	11
Mandatory training requirements	9
Training applies to all	2
Training applies to some	7
Applies to new premises	10
Applies to existing premises	6
Applies to on-sale premises	9
Applies to off-sale premises	7
Certification renewal period	1
Social Host	
Existence of law	24
Applies to underage parties	21
Civil violation	21
Criminal violation	18
Applies to a range of property types (residence, outdoor property)	22
Existence of a knowledge requirement	6
Window Advertising Limitations	
Existence of law	19
Distance provision from schools or parks	0
Establishment type specification	17
Outdoor/Billboard Advertising Limitations	
Existence of law	26
Prohibited near schools	10
Public Drinking Prohibitions	
Existence of law	50
Prohibitions across range of spaces (parks, beaches, etc.)	48
Narrow range of circumstances in which alcohol is permitted	46
Special Outdoor Events Restrictions	
Existence of law	44
Controlled alcohol consumption spaces	10
Security measures	13

Of the remaining policy topics, these 50 California cities display a mixed effort in terms of efficacy. With respect to the policy topic most addressed by these cities, public drinking, we find the highest adoption of important provisions recommended to reduce alcohol consumption in public [13]. A related policy topic, special outdoor events restrictions, shows a somewhat different pattern. Best practices suggest that providing security measures for events with alcohol availability can be an effective tool for reducing underage drinking [11,13] but only 13 of the 44 cities with events restrictions explicitly address security. Outdoor advertising is only represented as a policy in 26 cities, and only one prohibits outdoor advertising near schools. Finally, 19 cities have ordinances regulating window advertising with respect to alcohol, but none of them address the issue of distance from schools or parks.

A second way to analyze these data is offered in Table 7 which shows the range of scores for each policy topic as well as the mean, median and standard deviation for each. Of the four most critical policy topics, details additional to those available in Table 7 reveal the following: on the conditional use permit policy topic, scores ranged from 0 – 7, but only six of the forty-four CUP cities earned a score of 7. For deemed approved ordinances, the range was 0 – 6 and only two cities of the thirteen earned a score of 6. The social host range was 0 – 8, but none of the twenty-four cities earned a score of 8. With respect to responsible beverage service, the range was 0 – 10, but only one city out of the eleven with such policies earned a score of 10.

**Table 7:** Policy scoring ranges per policy topic

**Policy topic**	**Range**	**Median**	**Mean**	**Standard deviation**
CUPs	0–7	4.00	3.50	2.40
Deemed Approved Ordinances	0–6	0	1.02	1.82
Social Host	0–8	0	2.84	3.04
RBS	0–10*	0	1.48	2.89
Window Advertising	0–4	3.00	0.86	1.20
Outdoor/Billboard Advertising	0–2	2.00	0.72	0.75
Public Drinking	0–3	3.00	2.88	0.43
Special Outdoor Events	0–3	1.00	1.34	0.84

*RBS scores range from zero to ten because some categories are mutually exclusive.

In general, what is clear from the best practices scores across the 50 California cities is that much more can be done to address issues of harm associated with use and abuse of alcohol, including underage alcohol use. Most importantly, two policies often highlighted in best practices recommendations [2,13,57]–deemed approved ordinances and RBS training ordinances -- are uncommon across these California cities. Further, social host ordinances are present in only 24 of the 50 cities, suggesting that at least half of the cities do not employ these methods to limit alcohol availability to young people from social sources (e.g., friends, family members) in private settings. Even within policies that have higher adoption rates across cities, central elements are missing across many or most cities. A key example concerns the failure of 13 of the 44 cities with CUPs to prohibit or limit outlets in areas of over-concentration and high crime.

## Discussion and illustration of use of local datasets

Our study clarifies how local level policy research that is nuanced and specific can be achieved even absent comprehensive legal research tools such as those available at the U.S. state and federal levels. Remaining questions begin with the ways in which such data can be useful. We posit that there are several types of research studies that this dataset or similar datasets compiled by others can inspire. First, impact studies of the effect of differences in policies within or across jurisdictions that use the law as an independent variable compose one type. For example, localities with certain laws or combination of laws can be compared with localities without these laws on outcome factors such as crash fatalities. Another possibility is the use of before and after studies to analyze the impact of changing the law on localities.

A second type of study pertains to delineations of the scope of the law in one or more of these policy topics. Such research might focus on ordinances or regulations and case law to report on how both legislative and judicial action shape the way the law is carried out.

Assessments of determinants of policy choice by legislative bodies or by trial and appellate courts are a third research option. Studies in either branch of government may use policy as a dependent variable rather than an independent variable.

To illustrate how these data may be used for further research, we offer an analysis of the association between one of our eight policy topics (window advertising) and bar density, a measure of alcohol availability and a proxy measure for alcohol-related problems such as violence and DUI. This type of research falls into the first category of possible uses. Based on the hypothesis that policy adoption may be fueled by and indicate a variety of circumstances within cities beyond best practices recommendations, such as the presence of particularly difficult problems, we investigate the relationship between bar density and window advertising policies of cities. We expect to find a positive association between the two as greater bar density may be related to greater competition for customers. To test this relationship, we developed a multivariate research model of the correlates of density (the number of bars per roadway mile) in relationship to the window advertising policy with a range of control variables including demographic indicators such as population level, racial composition, household income, and employment levels in cities, and one measure of enforcement levels.

With respect to selection of control variables, much social science research indicates that an array of demographic, political, and structural variables of cities may affect the likelihood that trends in political adoption will be embraced. These include, but are not limited to, population size and the rural or urban nature of the city, the political ideology of citizens as well as their levels of income and education, their marital status, number of children, age range, race/ethnicity, religiosity, and party identification, the majority party status of city council (if partisan), presence or absence of an election year, and city government type. In U.S. cities, the two major government variations are mayor-council or council-manager in which either the mayor is elected separately from the city council or chosen from among city council representatives [75]. In this case, we focused on demographic variables that have been identified in the research literature as potential correlates of alcohol policies and/or outlet density, including population size, ethnic composition, median household incomes and unemployment rate [40,43,76]. City demographic data were obtained from the 2010 U.S. Census GeoLytics data [77].

Any model of public policy intended to reduce underage drinking must also include enforcement efforts. Although alcohol enforcement data on the local level tend to be difficult to secure, we rely on data representing state grant funding to cities from the California Alcohol Beverage Control Agency (CA ABC). The monies fund enforcement activities such as reducing alcohol sales to underage persons, enforcement of minor in possession laws, cops-in-shops programs targeting youth purchase, and shoulder tap interventions targeting adults purchasing alcohol for minors. Although cities have other avenues of enforcement funding such as city budgets and federal programs, these data provide a measure of the priority cities place on alcohol enforcement since they have to compete for these state funds (see Table 8 for total funds received by cities from the CA ABC in 2008–09, 2009–10, and 2010–11). Only 15 of the 50 cities were granted CA ABC funds in at least one of those years and funding levels ranged from $11,536 to $200,000. To account for the possible influence of city population size on levels of funding, the per capita funding rate was calculated (i.e., total amount of funding/city population size). Per capita funding rates ranged from 0.15 to 1.73 across cities.

**Table 8:** Funding from the California alcoholic beverage control agency to cities from 2008–2010

**City**	**Total gap funding (in U.S. dollars) 2008–2010**	**Per capita 2008–10**
01. Antioch	.0	.00000000
02. Bakersfield	.0	.00000000
03. Baldwin Park	.0	.00000000
04. Chico	.0	.00000000
05. Corona	43,222.00	.29086724
06. Davis	.0	.00000000
07. Diamond Bar	.0	.00000000
08. Fairfield	98,378.00	.92425780
09. Folsom	39,294.00	.55329630
10. Fresno	150,000.00	.30247240
11. Gardena	.0	.00000000
12. Hemet	.0	.00000000
13. Huntington Beach	100,000.00	.49387595
14. Huntington Park	.0	.00000000
15. La Mesa	.0	.00000000
16. Lake Forest	11,536.00	.14724803
17. Lancaster	62,528.00	.43100762
18. Livermore	.0	.00000000
19. Merced	.0	.00000000
20. Milpitas	.0	.00000000
21. Modesto	200,000.00	.95198200
22. Napa	.0	.00000000
23. National City	71,594.00	1.26665720
24. Orange	.0	.00000000
25. Petaluma	100,000.00	1.73193160
26. Pico Rivera	.0	.00000000
27. Rancho Cucamonga	.0	.00000000
28. Redding	.0	.00000000
29. Redlands	.0	.00000000
30. Richmond	.0	.00000000
31. Sacramento	100,000.00	.20785830
32. Salinas	.0	.00000000
33. San Buenaventura (Ventura)	.0	.00000000
34. San Leandro	.0	.00000000
35. San Rafael	.0	.00000000
36. Santa Barbara	98,831.00	1.09437700
37. Santa Clarita	.0	.00000000
38. Santa Cruz	.0	.00000000
39. Santa Maria	.0	.00000000
40. Santa Monica	.0	.00000000
41. Santa Rosa	.0	.00000000
42. Simi Valley	50,000.00	.39741206
43. Stockton	79,780.00	.27471600
44. Sunnyvale	.0	.00000000
45. Temecula	76,262.00	.74326540
46. Tracy	.0	.00000000
47. Turlock	.0	.00000000
48. Visalia	.0	.00000000
49. Vista	.0	.00000000
50. Walnut Creek	.0	.00000000
Total	$1,281,425.00	1.73193160

Data come from the California Alcohol Beverage Control Agency and U.S. Census Bureau.

Table 9 shows the results of an OLS regression model. Controlling for demographic variables and enforcement funds from the CA ABC, as hypothesized, there is a statistically significant positive relationship between the window advertising restrictions across cities and bar density within them. That is, the greater the bar density across cities, the higher the window advertising score.

**Table 9:** Testing the data: bar density as a function of window advertising policy

**Model**	**Unstandardized coefficients**		**Standardized coefficients**	**t**	**Sig.**
	**B**	**Std. Error**	**Beta**		
(Constant)	.106	.053		2.017	.050
Window Advertising Total	.012	.004	.423	3.035	.004
PER CAPITA 2008–10	.020	.012	.221	1.699	.097
Totalpop	−1.807E-8	.000	−.041	−.291	.773
PctWhite	.006	.031	.029	.199	.844
Median HH income	−7.975E-7	.000	−.322	−1.577	.122
Percentage of unemployees (both males and females) (unemployed/labor force)	−.414	.285	−.318	−1.451	.154

R^2^ = .28.

Dependent Variable = Bar density.

Independent Variables = Per capita enforcement funds from 2008–2011; total population, percentage white residents, median household income, and employment levels of cities.

## Conclusion: toward improved local alcohol policies to reduce underage drinking

In this study, we sought to expand the scope of research on local-level alcohol laws directed toward reduction in youth drinking in two ways. First, we demonstrated that it is possible to construct a reliable, valid, and comparative dataset on local-level alcohol policy ordinances across multiple policy topics, useful for a host of research designs. Because alcohol policies are important to reducing underage drinking and its resultant harms, this is good news for the research community.

Our second goal was to evaluate the presence, comprehensiveness (number of provisions), and stringency (restrictiveness) of eight local alcohol policies in 50 diverse California cities. Based on our data, it is clear that progress has been made since the mid-1980s. For example, during that time period, [Wittman and Hilton 37] found that less than a third of California cities had conditional use permit ordinances. Today 44 of the cities in our sample (or 88%) had CUP ordinances. Although effective dates for each type of ordinance in each city are not available as these data are inconsistently retained, it is clear that the policy work by cities to reduce underage drinking has accelerated over time. Mothers Against Drunk Driving (MADD) documents the recent sharp increase in social host liability ordinance in recent years [78]. The data provided here suggest, however, that more work needs to be done. Beyond adopting new ordinances, the clearest finding from this research is that existing ordinances across many policy areas can be strengthened. The combined best practices analysis and research findings provide ample guidance for such endeavors.

Finally, we explore how data of this type can be used, and provide an illustration of the impact of policy choices on alcohol-related outcomes. This effort is, without question, only an illustration of how these and related legal data can be used to assess the impact of local-level alcohol policies and our intent is to spur additional research of this type. This article suggests that such efforts are likely to be worthwhile.

## Competing interests

None of the authors has a financial or other competing interest.

## Authors’ contributions

Each of the authors contributed toward study design and methodological choices; ST, RT, and CC were responsible for design and execution of the legal data specification and collection; CC was responsible for design of the Access database with which data were recorded as well as initial data transformation and analysis. ST drafted the submission and each author reviewed and edited the final manuscript. All authors read and approved the final manuscript.
